# Xuebijing injection alleviates septic acute kidney injury by modulating inflammation, mitochondrial dysfunction, and endoplasmic reticulum stress

**DOI:** 10.1080/0886022X.2025.2483986

**Published:** 2025-03-27

**Authors:** Lei Zhang, Guangyuan Zhang, Weipu Mao, Si Sun, Shuchun Tao, Yue Gao, Nieke Zhang, Guiya Jiang, Ming Chen, Xun Lu, Shuqiu Chen

**Affiliations:** a Department of Urology, Zhongda Hospital, Southeast University, Nanjing, PR China; b Institute of Urology, Surgical Research Center, School of Medicine, Southeast University, Nanjing, PR China; c Department of Urology, Children’s Hospital of Nanjing Medical University, Nanjing, PR China

**Keywords:** Traditional Chinese herb, sepsis, toll like receptor 4, network pharmacology

## Abstract

**Background:**

Xuebijing (XBJ) injection has been used to treat sepsis. However, the effect and mechanism of XBJ injection in the treatment of septic acute kidney injury (AKI) is unknown. This study aimed to explore the therapeutic effect of XBJ injection on septic AKI and elucidate its possible mechanisms.

**Methods:**

Network pharmacological analysis was conducted using databases of GeneCards, TCMSP, SwissTargetPrediction and STRING. *In vivo*, a septic AKI model was established in C57BL/6 mice by cecal ligation and puncture (CLP). The groups were Sham, XBJ, CLP, and CLP + XBJ (10 mL/kg IV) (*n* = 5). Tubular damage, renal function, and levels of inflammation and apoptosis in the kidneys were evaluated. *In vitro* model was lipopolysaccharide (LPS, 100 μg/mL) stimulated HK-2 cells. The groups were PBS, XBJ, LPS, and LPS + XBJ (XBJ injected at 10 dilutions). Cell viability, apoptosis, inflammation, mitochondrial function and, endoplasmic reticulum (ER) stress were also assessed.

**Results:**

Network pharmacological analysis identified Toll like receptor 4 (TLR4) as the core gene in XBJ against septic AKI, and the inflammatory response was the most enriched pathway. XBJ treatment significantly alleviated tubular damage in CLP mice by down-regulating serum creatinine (SCr), blood urea nitrogen (BUN), kidney injury molecule 1 (KIM1), and neutrophil gelatinase-associated lipocalin (NGAL). Furthermore, both *in vivo* and *in vitro* experiments demonstrated that XBJ treatment could inhibit apoptosis, inflammation, mitochondrial dysfunction, and ER stress *via* TLR4/MyD88/NF-κB axis.

**Conclusion:**

This study indicates that XBJ injection is a promising drug for the treatment of septic AKI.

## Introduction

Sepsis, characterized by an uncontrolled response to serious infections, is one of the leading causes of death in critically ill patients worldwide [1,2]. The incidence of acute kidney injury (AKI) is approximately 50% among patients with sepsis, which not only increases the mortality of patients with sepsis, but also contributes to the development of chronic kidney disease [3–6]. Currently, the treatment of sepsis mainly focuses on preventing organ dysfunction. However, many studies exploring therapeutic targets for sepsis have failed, and the mortality rate remains high among septic patients [7–10]. Therefore, it is crucial to develop both early diagnostic strategies [11] and effective therapeutic interventions [12,13] for the treatment of septic AKI.

Xuebijing (XBJ) injection is a combination of five traditional Chinese herbs: *Paeonia lactiflora* Pall (Paeoniaceae, Radix Rubra, Chi Shao), *Angelica sinensis* (Oliv.) Diels (Apiaceae, Radix, Dang Gui), *Ligusticum chuanxiong* Hort. (Apiaceae, Chuanxiong Rhizoma, Chuan Xiong), *Carthamus tinctorius* L. (Asteraceae, Carthami Flos, Hong Hua), and *Salvia miltiorrhiza* Bge. (Lamiaceae, Radix Et Rhizoma, Dan Shen) [14]. Numerous studies have reported the anti-inflammatory, immunomodulatory, and coagulation functions [15–18]. A recently published multi-center randomized clinical trial showed that, compared with placebo, XBJ administration reduced 28-day mortality without increasing adverse effects in patients with sepsis [19].

Recently, traditional Chinese medicine (TCM) has attracted increasing attention as a treatment septic AKI. For example, Zhang et al. [20] reported that arbutin extracted from *Chinese yam* alleviated sepsis-induced AKI in an LPS-induced sepsis model. Pan et al. [21] suggested that salidroside, extracted from the traditional Chinese herb *Rhodiola rosea* (Crassulaceae, L.), exerts a reno-protective effect by activating the sirtuin 1 (SIRT1)/nuclear factor erythroid 2-related factor 2 (Nrf2) pathway during sepsis. Furthermore, Yang et al. [22] showed that neohesperidin dihydrochalbazone derived from chalcones attenuated AKI by inhibiting oxidative stress, inflammation, and apoptosis through the p38 mitogen-activated protein kinase (MAPK) pathway. However, whether XBJ injection can alleviate septic AKI and the underlying mechanisms need to be further elucidated.

Here, we report that XBJ injection alleviates septic AKI by modulating inflammation, mitochondrial dysfunction, and endoplasmic reticulum (ER) stress.

## Material and methods

### Network pharmacologic analysis

Genes related to septic AKI were obtained from the online GeneCards database (http://www.genecards.org). The active components of XBJ were screened from the TCMSP database (https://old.tcmsp-e.com/tcmsp.php) under the conditions of bio-availability > 30 and drug-likeness > 0.18. The SwissTargetPrediction database (http://www.swisstargetprediction.ch/) was used to predict the possible targets of the XBJ components. The protein-protein interaction (PPI) was constructed using the STRING database (https://string-db.org/cgi/input.pl). Gene Ontology (GO) functional annotation and Kyoto Encyclopedia of Genes and Genomes (KEGG) pathway analysis were performed using the Xiantao academic tool (https://www.xiantaozi.com/).

### Reagents

LPS (L2880) from *Escherichia coli* 055: B5 (> 99% purity) was purchased from Sigma-Aldrich (St. Louis, MO, USA). The XBJ injection (10 mL/ampoule) was provided by Tianjin Hongri (Tianjin, China).

### Animals

Male C57BL/6 mice (RRID: MGI:2159769), aged six to eight weeks and weighing 20-22 g, were obtained from Vital River Laboratories (Nanjing, China). Male mice were chosen to minimize variability due to hormonal fluctuations in female mice, which could affect sepsis modeling and treatment outcomes. The mice were housed under standard laboratory conditions with a 12-h light/dark cycle, controlled temperature (22 ± 2 °C), and humidity (50 ± 10%), with *ad libitum* access to food and water.

To establish the murine sepsis model, cecal ligation and puncture (CLP) was performed following the protocol [23]. Sham-operated mice underwent anesthesia and midline laparotomy, but without cecal ligation or puncture. XBJ injection (10 mL/kg) was administrated to mice *via* tail vein injection 12 h after CLP as a single dose. The animals were randomly divided into four groups: sham, XBJ, CLP, and CLP + XBJ, each consisting of five mice for complete sample collection and data analysis. 24 h post-CLP, kidneys and blood samples were collected for further examination. For survival analysis, an additional cohort of mice was used, where each group contained 5 mice. The survival rate was monitored over a 72-h period, and Kaplan-Meier survival curves were plotted to compare the survival rates between groups.

The sample size was determine using the resource equation method, ensuring adequate statistical power while adhering to the principles of the 3Rs (Replacement, Reduction, Refinement). The formula used was E = Total number of animals - total number of groups, where E should be between 10 and 20. In our study, *E* = 20 − 4 = 16, which is within the acceptable range.

To minimize suffering, mice were monitored every 4 h for signs of severe distress, pain, or morbidity, in accordance with the NC3Rs guidelines on Humane Endpoints and the American Veterinary Medical Association Guidelines for the Humane Slaughter of Animals. Humane endpoints were determined based on specific behavioral observations, including severe lethargy, inability to eat or drink, severe respiratory distress, and unresponsiveness to gentle stimuli.

Euthanasia was performed by first anesthetizing the mice through inhalation of an overdose of isoflurane, inducing deep central nervous system depression to prevent pain and distress. After achieving adequate anesthesia, carbon dioxide (CO_2_) was introduced to ensure a humane reduction in consciousness. Finally, cervical dislocation was performed to confirm death, verified by the cessation of cardiac and respiratory activity. Blood samples were collected from the retro-orbital sinus under anesthesia using a capillary tube and kidneys were resected by Dr. Lei Zhang before euthanasia.

All animal experiments were approved by the Laboratory Animal Care and Welfare Committee of Southeast University (Approval No. 20231124001), and *in vivo* experiments were conducted by Dr. Lei Zhang, Dr. Guangyuan Zhang, and Dr. Si Sun.

### Renal function analysis

To collect serum, mice blood samples from different groups were centrifuged at 4 °C of 3000 rpm for 15 min. Serum creatinine (SCr) and blood urea nitrogen (BUN) levels were measured using commercial assay kits (C011-2-1, and C012-2-1, Jiancheng, Nanjing, China).

### Histologic examination

Mouse kidney tissues were fixed in 4% paraformaldehyde (Servicebio, Wuhan, China) and embedded in paraffin. Tubular injury was determined by periodic acid-Schiff (PAS) staining. Scores reflecting tubular injury were calculated as previously described [24]. Scores of 0, 1, 2, 3, and 4 indicate < 10%, 10-25%, 26-50%, and 51-75% injured tubules, respectively.

### Immunohistochemistry

Kidney sections were dewaxed in xylene and rehydrated through a graded ethanol series. Antigen retrieval was performed by boiling the sections in 10 mM sodium citrate solution (pH 6.0) using a microwave at medium power for 10 min. After cooling to room temperature, the sections were washed with phosphate-buffered saline (PBS). To block nonspecific binding, sections were incubated with 10% goat serum for 1 h at room temperature. Following blocking, the sections were incubated overnight at 4 °C with primary antibodies: anti-F4/80 (Rabbit, 1:500, GB11027, RRID: AB_2814687) and anti-Ly6G (Rabbit, 1:500, GB11229, RRID: AB_2814689), both sourced from Servicebio (Wuhan, China). The next day, sections were washed three times with PBS and then incubated with secondary antibody (S-vision Immunohistochemistry Kit, G1313-100T, Servicebio) for 20 min at room temperature. Color development was achieved using DAB working solution, followed by counterstaining with hematoxylin. Sections were dehydrated and mounted with a coverslip. Images were captured using a light microscope. The number of positive F4/80 and Ly6G cells was quantified using ImageJ software (version 1.51, USA) by selecting representative fields and calculating the average number of positive cells per field.

### TUNEL staining

Apoptotic cells in kidney sections were measured by terminal deoxynucleotidyl transferase mediated dUTP nick-end labeling (TUNEL) assay (S7110, Millipore, USA). Briefly, paraffin-embedded kidney sections were deparaffinized in xylene and rehydrated through a series of graded ethanol washes. Sections were then permeabilized with proteinase K for 15 min at room temperature. After washing with PBS, sections were incubated with a TUNEL reaction mixture containing terminal deoxynucleotidyl transferase (TdT) and labeled dUTP in a humidified chamber at 37 °C for 1 h in the dark. The reaction was stopped by washing with PBS. Sections were counterstained with DAPI (G1012, Servicebio) to visualize nuclei and mounted with a coverslip. TUNEL-positive cells were visualized and counted under a fluorescence microscope (YHF39, Yuehe, Shanghai, China).

### Quantitative real-time PCR analysis

Total RNA was extracted from kidney tissues and cells using TRIzol reagent (R401-01, Vazyme, Nanjing, China) following the manufacturer’s instructions. The concentration and purity of the extracted RNA were assessed using a Nanodrop spectrophotometer. For reverse transcription, 1 μg of total RNA was reverse transcribed into cDNA using a reverse transcription kit (G3337, Servicebio) according to the manufacturer’s protocol. Quantitative real-time PCR was performed using a SYBR Green PCR kit (G3325, Servicebio). The cycling conditions were as follows: pre-denaturation at 95 °C for 30 s, followed by 40 cycles of denaturation at 95 °C for 10 s, annealing at 60 °C for 30 s, and extension at 72 °C for 30 s. Gene expression levels were normalized to GAPDH as an internal reference, and relative expression was calculated using the 2^-ΔΔCt^ method. All primers were listed in Table 1.

**Table 1.** Primer sequences used in real-time PCR analysis.

Gene	Forward Primer	Reverse Primer
M-GAPDH	GCATGGCCTTCCGTGTTC	GATGTCATCATACTTGGCAGGTTT
M-KIM1	ACATATCGTGGAATCACAACGAC	ACTGCTCTTCTGATAGGTGACA
M-NGAL	GCAGGTGGTACGTTGTGGG	CTCTTGTAGCTCATAGATGGTGC
M-Bcl-2	TGTGAGGACCCAATCTGGAAA	TTGCAATGAATCGGGAGTTG
M-Bax	GATCAGCTCGGGCACTTTAG	TTGCTGATGGCAACTTCAAC
M-IL-1β	TGCCACCTTTTGACAGTGATG	AAGGTCCACGGGAAAGACAC
M-IL-6	AAAGAGTTGTGCAATGGCAATTCT	AAGTGCATCATCGTTGTTCATACA
M-TNF-α	CATCTTCTCAAAATTCGAGTGACAA	TGGGAGTAGACAAGGTACAACCC
M-MCP-1	CTTCTGGGCCTGCTGTTCA	CCAGCCTACTCATTGGGATCA
M-PGC-1α	CACCAAACCCACAGAAAACAG	GGGTCAGAGGAAGAGATAAAGTTG
M-ATP5a-1	CATTGGTGATGGTATTGCGC	TCCCAAACACGACAACTCC
M-NDUFS8	GTTCATAGGGTCAGAGGTCAAG	TCCATTAAGATGTCCTGTGCG
M-TOM20	GCTAAGGAGAGAGCTGGGCTTT	TGGTCCACACCCTTCTCGTAGT
M-PERK	GCCGACGATCAAATGGAAGC	ACCTGACTGTGATCTGCGTG
M-IRE1α	CGGGAGAACATCACTGTCCC	GCTCGTGCCAGTAGTAGGTC
M-XBP1	GAACCAGGAGTTAAGAACACG	AGGCAACAGTGTCAGAGTCC
H-GAPDH	GGAAGCTTGTCATCAATGGAAATC	TGATGACCCTTTTGGCTCCC
H-Bcl-2	GGTGGGGTCATGTGTGTGG	CGGTTCAGGTACTCAGTCATCC
H-Bax	CCCGAGAGGTCTTTTTCCGAG	CCAGCCCATGATGGTTCTGAT

M, denotes mice; H, denotes human.

### Western blot analysis

Proteins were extracted from kidney tissues and cells using radioimmunoprecipitation assay (RIPA) buffer containing protease and phosphatase inhibitors. The supernatants were collected after centrifuging at 12,000 rpm for 20 min at 4 °C. Protein concentration was determined using a bicinchoninic acid (BCA) assay kit (KGB2101, Keygen Biotech, Nanjing, China), following the manufacturer’s instructions. Briefly, a standard curve was prepared using serial dilutions of bovine serum albumin (BSA) as the standard protein. For each sample, 20 μL of the protein extract was mixed with 200 μL of the BCA working reagent in a 96-well plate. The plate was incubated at 37 °C for 30 min. Absorbance was measured at 562 nm using a microplate reader. The protein concentrations of the samples were calculated by comparing their absorbance values to the standard curve, ensuring accurate quantification.

Western blot process was exerted according to our previous study [25]. Briefly, equal amounts of protein (30 µg per lane) were loaded onto SDS-PAGE gels and separated by electrophoresis. Proteins were then transferred to 0.2 μM polyvinylidene fluoride (PVDF) membranes (ISEQ85R, Millipore, MA, USA). Membranes was blocked with 5% BSA for 1 h at room temperature and then incubated with primary antibodies at 4 °C overnight. Then, membranes were washed and incubated with secondary antibodies for 1 h at room temperature. Proteins were visualized using an enhanced chemiluminescence (ECL) detection system (G2074, Servicebio). Densitometry analysis of protein bands was performed using ImageJ software to quantify protein expression levels relative to the loading control, β-actin.

The primary antibodies used in the study were as follows, with details including name, species, dilution, catalog number, RRID, and supplier: Anti-NGAL: Rabbit, 1:1000, ab125075, AB_10978084, Abcam (MA, USA); Anti-phospho-IRE1α: Rabbit, 1:1000, ab48187, AB_873899, Abcam; Anti-Bcl-2: Rabbit, 1:1000, #4223, AB_1903909, Cell Signaling Technology (MA, USA); Anti-Cleaved-caspase 3: Rabbit, 1:1000, #9664, AB_2070042, Cell Signaling Technology; Anti-Caspase 3: Rabbit, 1:1000, #9662, AB_331439, Cell Signaling Technology; Anti-ERK: Rabbit, 1:1000, #4695, AB_390779, Cell Signaling Technology; Anti-phosphor-ERK: Rabbit, 1:1000, #4370, AB_2315112, Cell Signaling Technology; Anti-eIF2α: Rabbit, 1:1000, #5324, AB_10692650, Cell Signaling Technology; Anti-phospho-eIF2α: Rabbit, 1:1000, #3398, AB_2096481, Cell Signaling Technology; Anti-IRE1α: Rabbit, 1:1000, #3294, AB_823548, Cell Signaling Technology; Anti-XBP1s: Rabbit, 1:1000, #12782, AB_2687943, Cell Signaling Technology; Anti-Bax: Rabbit, 1:5000, 50599-2-Ig, AB_2061561, Proteintech Group (Wuhan, China); Anti-BiP: Rabbit, 1:2000, 11587-1-AP, AB_2119855, Proteintech Group; Anti-β-actin: Rabbit, 1:5000, 81115-1-RR, AB_2923704, Proteintech Group; Anti-PGC-1α: Rabbit, 1:5000, 66369-1-Ig, AB_2828002, Proteintech Group; Anti-IL-6: Rabbit, 1:1000, 21865-1-AP, AB_11142677, Proteintech Group; Anti-p38: Rabbit, 1:2000, 14064-1-AP, AB_2878007, Proteintech Group; Anti-phospho-p38: Rabbit, 1:1000, 28796-1-AP, AB_2918205, Proteintech Group; Anti-IL-1β: Mouse, 1:1000, sc-12742, AB_627791, Santa Cruz Biotechnology (TX, USA); Anti-TNF-α: Mouse, 1:1000, sc-12744, AB_628372, Santa Cruz Biotechnology; Anti-MCP-1: Rat, 1:1000, sc-52701, AB_628867, Santa Cruz Biotechnology; Anti-NOX4: Mouse, 1:1000, sc-518092, AB_3186233, Santa Cruz Biotechnology; Anti-Nrf2: Mouse, 1:1000, sc-365949, AB_10917561, Santa Cruz Biotechnology; Anti-phospho-IκB: Mouse, 1:1000, sc-8404, AB_627773, Santa Cruz Biotechnology; Anti-IκB: Mouse, 1:1000, sc-1643, AB_627772, Santa Cruz Biotechnology; Anti-TOM20: Mouse, 1:500, sc-17764, AB_628381, Santa Cruz Biotechnology; Anti-TLR4: Rabbit, 1:1000, GB11519, AB_3186234, Servicebio; Anti-MyD88: Rabbit, 1:1000, GB111554, AB_3186235, Servicebio.

### Cell culture

Human renal tubular epithelial cells (HK-2) cells were obtained from the National Collection of Authenticated Cell Cultures (Catalog number: GNHu47, RRID: CVCL_0302, Shanghai, China,). These cells were maintained at 37 °C in a humidified atmosphere with 5% CO_2_ and cultured with Dulbecco’s Modified Eagle Medium/Nutrient Mixture F-12 (DMEM/F12) medium with 10% fetal bovine serum (FBS), 1% penicillin-streptomycin (Keygen Biotech). Cells were seeded at an appropriate density in culture flasks and allowed to reach 70–80% confluence before passaging. For experimental treatments, cells were seeded 24 h prior to ensure proper adhesion and entry into the logarithmic growth phase. Subsequently, they were treated with or without LPS and XBJ injection at appropriate concentrations and for an appropriate duration by direct addition to the culture medium.

### Cell viability analysis

Cell viability was determined using a Cell Counting Kit-8 assay (CCK-8, BS350A, Biosharp, China). HK-2 cells were seeded in 96-well culture plates at a density of 5000 cells/well. Cells were treated with LPS or XBJ for indicated time. After treatment, 10 μL CCK-8 reagent was added to each well, and the plate was incubated at 37 °C for 2 h. The absorbance at 450 nm was measured using a microplate reader. Cell viability was calculated as a percentage of the control (untreated) cells, using the formula: Cell viability = (Absorbance of treated cells/Absorbance of control cells) × 100.

### Immunofluorescence

After fixing with 4% paraformaldehyde, HK-2 cells were incubated with a nuclear factor-κB (NF-κB) antibody (1:2000, GB11997, RRID: AB_3083517, Servicebio) overnight at 4 °C. The cell nucleus was stained with DAPI (G1012, Servicebio). Immunofluorescent images were obtained using a fluorescence microscope (YHF39, Yuehe). To measure intracellular reactive oxygen species (ROS), 2,7-dichlorodihydrofluorescein diacetate (DCFH-DA, S0033S, Beyotime, Shanghai, China) was used to probe ROS in HK-2 cells. Briefly, DCFH-DA was dissolved in medium and co-cultured with HK-2 cells for 30 min. The medium was then removed, and the cells were rinsed with PBS. Finally, the HK-2 cells were observed under a fluorescence microscope. ROS accumulation in the kidneys was measured by staining with dihydroethidium (DHE, S0063, Beyotime) according to the manufacturer’s protocol. To measure the mitochondrial membrane potential, HK-2 cells were stained with a JC-1 kit (G1515, Servicebio). Briefly, HK-2 cells were incubated with 1 mL of JC-1 working buffer and observed under a fluorescence microscope. The red-to-green fluorescence intensity was calculated to indicate mitochondrial damage.

### Enzyme-linked immunosorbent assay (ELISA)

The concentrations of inflammatory cytokines in the supernatant of HK-2 cells were measured using commercial ELISA kits: interleukin-6 (IL-6) (E-EL-H6156, Elabscience Biotechnology, Wuhan, China); tumor necrosis factor-α (TNF-α) (E-EL-H0109, Elabscience Biotechnology); IL-1β (E-EL-H0149, Elabscience Biotechnology). Briefly, cell culture supernatants were collected and centrifuged to remove debris. Standards and samples were added to the ELISA plate wells and incubated for 2 h at room temperature. After washing, a biotinylated detection antibody was added, followed by streptavidin-HRP. The colorimetric reaction was developed using the substrate solution, and absorbance was measured at 450 nm using a microplate reader. Cytokine concentrations were calculated based on the standard curve generated for each cytokine.

### Flow cytometry analysis

Cells were harvested by trypsinization, washed twice with cold PBS, and resuspended in 1 × binding buffer at a concentration of 1 × 10^6^ cells/mL. Cell apoptosis was measured using an Annexin V/PI apoptosis detection kit (A211-02, Vazyme). For staining, 100 μL of the cell suspension (approximately 1 × 10^5^ cells) was transferred to a flow cytometry tube. Cells were incubating with 5 μL Annexin V-FITC and 5 μL PI at room temperature for 15 min in the dark. Subsequently, 400 μL of 1 × binding buffer was added to each tube. Flow cytometry was performed using flow cytometry (Attune NxT, Thermo Fisher Scientific, USA). The excitation wavelength of the flow cytometer is 488 nm. The green fluorescence of FITC is detected in the BL1 channel (530/30 nm filter) and the red fluorescence of PI is detected in the BL2 channel (574/26 nm filter). Cells were initially gated based on forward scatter (FSC) and side scatter (SSC) to exclude debris and aggregates. A live cell gate was established to differentiate between Annexin V-FITC-positive/PI-negative (early apoptotic) and Annexin V-FITC-positive/PI-positive (late apoptotic/necrotic) populations. Doublets were excluded by plotting FSC-area against FSC-height. Data were analyzed using FlowJo software (version 10.6.2), and the percentage of cells in each quadrant was calculated to determine the levels of apoptosis.

### Transmission electron microscopy

To detect the renal ER structure, fresh mouse kidneys were fixed in cold 2.5% glutaraldehyde and processed routinely. The morphology of the ER was captured using TEM (HITACHI H7650 TEM; Tokyo, Japan) at ×15,000 magnification.

### Measurement of oxidative stress indices

Specific assay kits were used to determine malondialdehyde (MDA, A003-1-1, Jiancheng), superoxide dismutase (SOD, A001-3-2, Jiancheng), and glutathione peroxidase (GSH-Px, A005-1-2, Jiancheng) levels in HK-2 cells and mouse kidney tissues according to the manufacturer’s protocol.

### Statistical analysis

Data are presented as mean ± standard deviation and were analyzed using GraphPad Prism 7.01 (GraphPad Software, San Diego, CA, USA). Data normality was assessed using the Shapiro-Wilk test before applying parametric tests. All experiments were repeated at least three times. Statistical significance between two groups was determined using a two-tailed Student’s *t*-test (or the Mann-Whitney U test for non-normally distributed data). Differences among multiple groups were determined using one-way or two-way analysis of variance (ANOVA), followed by Tukey’s post-hoc test when applicable. For non-parametric comparisons among multiple groups, the Kruskal-Wallis test was used. The overall survival rate of mice was calculated using the Kaplan–Meier method and analyzed using the log-rank test. Statistical significance was set at *p <* 0.05.

## Results

### Network pharmacological analysis of XBJ for the treatment of septic AKI

**Figure 1.**
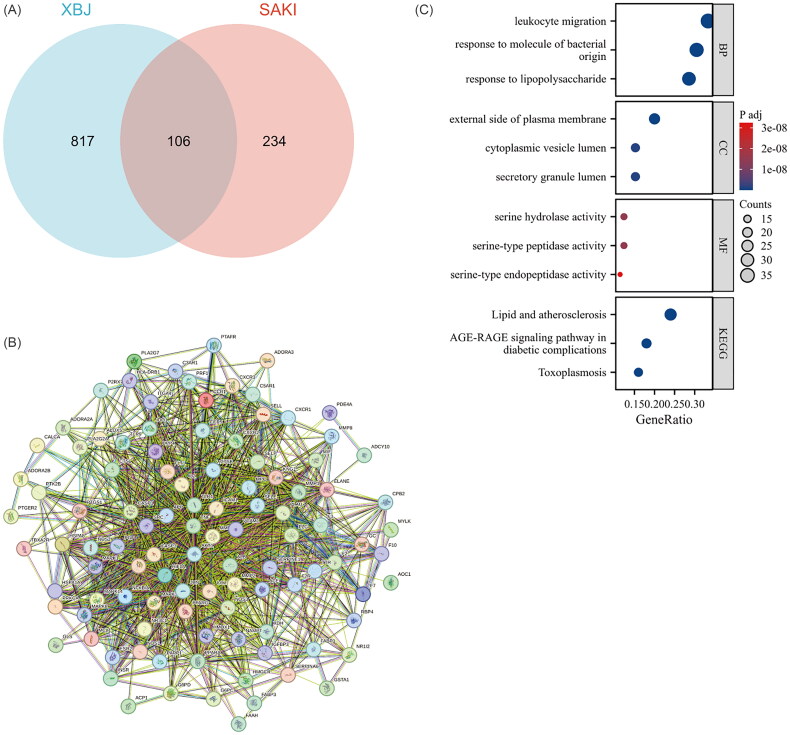
Network pharmacological analysis of XBJ for treatment of septic AKI. (A) The Venn diagram of XBJ and septic AKI. (B) PPI network. (C) Gene ontology (GO) functional annotation and Kyoto encyclopedia of genes and genomes (KEGG) pathways analysis of XBJ for septic AKI.

The mechanism of action of natural drugs is relatively complex and may involve multiple targets and pathways. Accordingly, we conducted a network pharmacology study to explore the possible targets and mechanisms of action of XBJ in septic AKI. We identified 340 genes related to septic AKI from an online database (GeneCards) and 923 XBJ-related genes from a drug database (TCMSP). We identified 106 core genes in the septic AKI and XBJ treatment groups by defining the interaction between the two groups (Figure 1(A)). To visualize the defined core genes, we constructed a PPI network and found that many inflammatory-related targets, such as toll like receptor 4 (TLR4), TNF-α and NF-κB were located at the core of the network (Figure 1(B)). Additionally, we identified that the enrichment scores of leukocyte migration, response to molecules of bacterial origin, and response to lipopolysaccharides were relatively high in biological processes (Figure 1(C)), indicating that these processes might play a vital role in XBJ against septic AKI. These results suggested that the therapeutic effect of XBJ against septic AKI was mediated mainly by an inflammatory response.

### XBJ injection alleviated CLP-induced AKI

**Figure 2.**
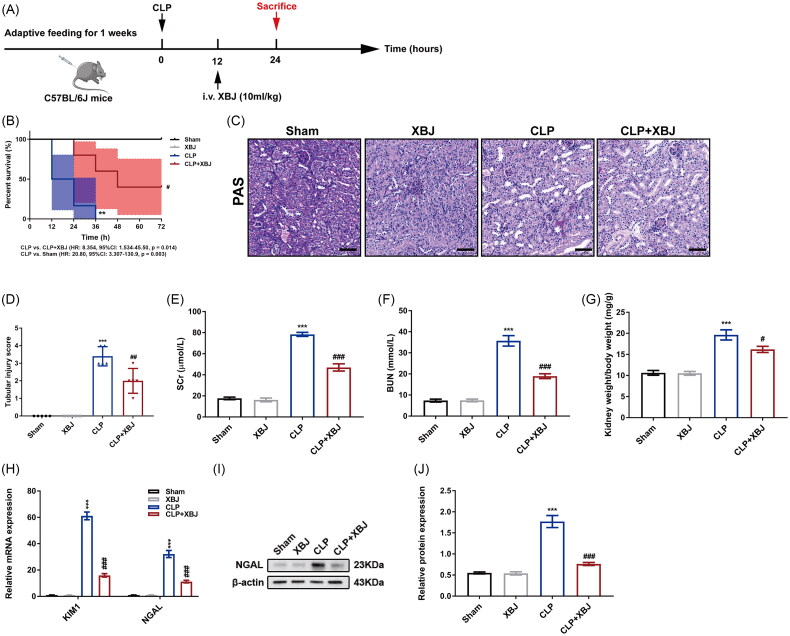
XBJ injection alleviated CLP-induced AKI. (A) The protocol of animal experiments of XBJ treatment in CLP mice. (B) The survival analysis of mice in different groups (*n* = 5). (C) Representative PAS staining images of kidneys (scale bar = 50 μm). (D) Scores of tubular injury in kidneys (*n* = 5). (E) SCr and (F) BUN levels (*n* = 5). (G) Kidney weight to body weight index of mice (*n* = 5). (H) The mRNA expression levels of KIM1 and NGAL in kidneys (*n* = 3). (I) The protein level of NGAL in kidneys detected by western blot and (J) quantified by densitometry (*n* = 3). ***p < 0.001 for CLP versus sham; ^#^*p* < 0.05, ^##^*p* < 0.01, ^###^*p* < 0.001 for CLP + XBJ versus CLP.

To evaluate the protective effect of XBJ injection on sepsis, mice were randomly divided into four groups: sham, XBJ, CLP, and CLP + XBJ. XBJ was injected at 10 mL/kg 12 h after CLP, according to a previous study [18] (Figure 2(A)). As shown in Figure 2(B), XBJ treatment significantly improved the survival rate of CLP mice during the 72-h follow-up (median survival: 48 h *vs.* 18 h, *p* = 0.014). However, XBJ had no impact on the sham group, indicating the safety of XBJ injection *in vivo*. Therefore, we investigated the reno-protective effects of XBJ extract. Compared with the sham group, CLP induced significant tubular injury, as revealed by PAS staining, which represented significant tubular damage with dilatation and swelling of tubules, formation of casts, and absence of a tubular brush border. XBJ treatment significantly ameliorated tubular damage in CLP mice (Figure 2(C) and (D)). Moreover, XBJ administration markedly improved renal function in CLP mice, as decreased levels of SCr (78.37 ± 1.93 *vs.* 46.99 ± 3.46 μmol/L, *p* < 0.001) and BUN (35.71 ± 2.46 *vs.* 18.95 ± 1.15 mmol/L, *p* < 0.001) (Figure 2(E) and (F)). The XBJ injection also decreased the kidney index (kidney weight to body weight) in CLP mice (Figure 2(G)). In addition, XBJ decreased the levels of kidney injury markers kidney injury molecule 1 (KIM1) and neutrophil gelatinase-associated lipocalin (NGAL), as revealed by qPCR and western blot (Figure 2(H–J)). Overall, these data demonstrate that XBJ injection ameliorated CLP-induced kidney injury.

### XBJ injection inhibited inflammation and apoptosis in CLP-injured kidneys

**Figure 3.**
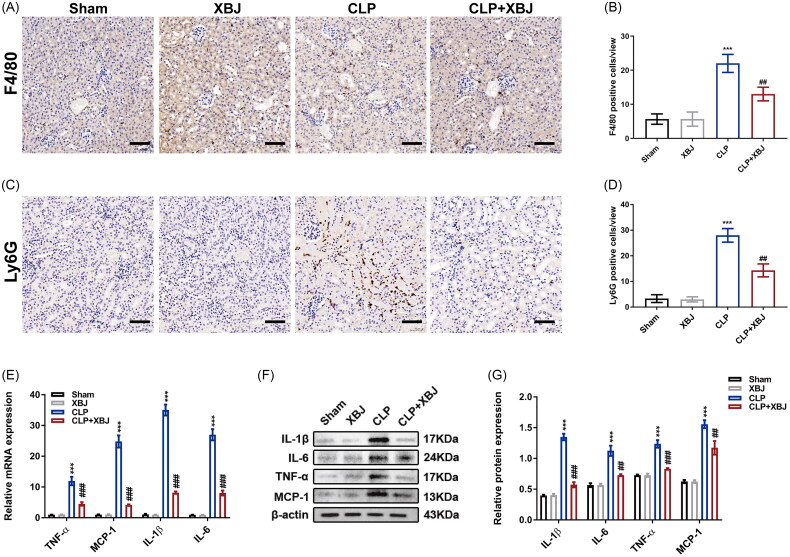
XBJ injection inhibited inflammation in CLP-injured kidneys. (A) Representative images of F4/80 staining in the renal cortex (scale bar = 50 μm) and (B) the quantification of F4/80 positive cells in kidneys (*n* = 5). (C) Representative images of Ly6G staining in the renal cortex (scale bar = 50 μm) and (D) the quantification of Ly6G positive cells in kidneys (*n* = 5). (E) The mRNA expression levels of IL-1β, IL-6, MCP-1, and TNF-α in kidneys (*n* = 3). (F) The protein levels of kidney IL-1β, IL-6, MCP-1, and TNF-α detected by Western blot and (G) quantified by densitometry (*n* = 3). ***p < 0.001 for CLP versus sham; ^#^*p* < 0.05, ^##^*p* < 0.01, ^###^*p* < 0.001 for CLP + XBJ versus CLP.

**Figure 4.**
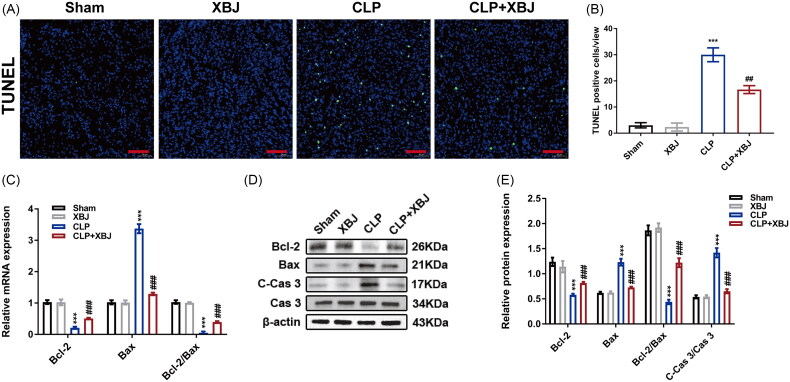
XBJ injection decreased apoptosis in CLP-injured kidneys. (A) Representative TUNEL staining images of kidney cortex (scale bar = 50 μm) and (B) quantification of TUNEL positive cells in kidneys (*n* = 5). (C) The mRNA expression levels of Bcl-2, Bax and Bcl-2/Bax in kidneys (*n* = 3). (D) The protein levels of kidney Bcl-2, C-Cas 3, Cas 3, and Bax and the (E) quantification analysis (*n* = 3). ***p < 0.001 for CLP versus sham; ^##^*p* < 0.01, ^###^*p* < 0.001 for CLP + XBJ versus CLP.

Notably, inflammation and apoptosis are characteristic of septic AKI [26–28]. Therefore, we assessed the effects of XBJ on inflammation and apoptosis *in vivo*. As shown in Figure 3(A–D), CLP initiated the accumulation of macrophages and neutrophils in the kidneys, which was mitigated by XBJ treatment. Furthermore, XBJ treatment inhibited the inflammatory response as evidenced by decreased mRNA and protein levels of pro-inflammatory cytokines of IL-1β, IL-6, monocyte chemoattractant protein-1 (MCP-1), and TNF-α in CLP injured kidneys (Figure 3(E–G)). To illustrate the role of XBJ in kidney apoptosis, TUNEL assay was performed to detect apoptotic cells in the kidneys. As shown in Figure 4(A) and (B), XBJ treatment dramatically decreased TUNEL positive cells in CLP-injured kidneys. Additionally, XBJ significantly decreased the levels of the pro-apoptotic markers cleaved-caspase 3 (C-Cas 3) and Bcl-2-associated X protein (Bax), while increasing the expression of the anti-apoptotic marker B-cell lymphoma 2 (Bcl-2) (Figure 4(C–E)). Overall, these results imply that XBJ injection inhibits inflammation and apoptosis in CLP injured kidneys.

### XBJ injection reduced inflammation and apoptosis in LPS-stimulated HK-2 cells

**Figure 5.**
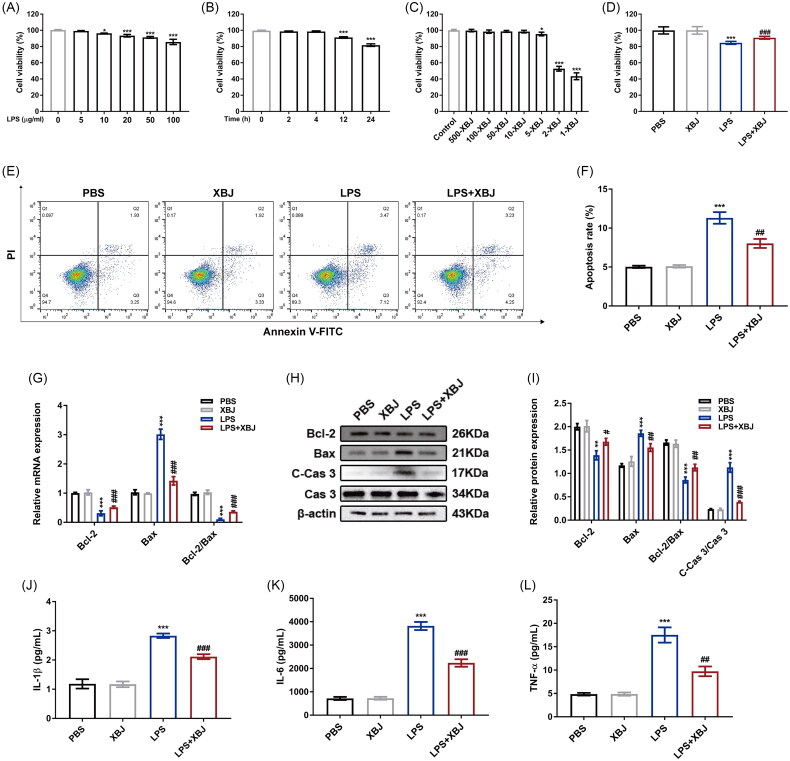
XBJ injection inhibited apoptosis and inflammation in LPS-stimulated HK-2 cells. (A) The cell viability of HK-2 cells under the stimulation of LPS (0, 5, 10, 20, 50, and 100 μg/mL) for 24 h (*n* = 3). (B) The cell viability of HK-2 cells under the stimulation of LPS (100 μg/mL) for indicated times (0, 2, 4, 12, 24 h) (*n* = 3). (C) The cytotoxic effect of XBJ (at a dilution of 500-, 100-, 50-, 10-, 5-, 2- and 1-fold) on HK-2 cells (*n* = 3). (D) The cytoprotective effect of XBJ on LPS-treated HK-2 cells (*n* = 3). (E) Representative flow cytometric plots and (F) quantification of apoptosis in HK-2 cells (*n* = 3). (G) The mRNA expression levels of Bcl-2 and bax in HK-2 cells (*n* = 3). (H) The protein levels of Bcl-2, C-cas 3, cas 3, and bax in HK-2 cells detected by Western blot and (I) quantified by densitometry (*n* = 3). (J) The quantification of IL-1β in the supernatants of HK-2 cells (*n* = 5). (K) The quantification of IL-6 in the supernatants of HK-2 cells (*n* = 5). (L) The quantification of TNF-α in the supernatants of HK-2 cells (*n* = 5). **p < 0.01, ***p < 0.001 for LPS versus PBS; ^#^*p* < 0.05, ^##^*p* < 0.01, ^###^*p* < 0.001 for LPS + XBJ versus LPS.

To mimic sepsis *in vitro*, we established a cellular model using LPS to stimulate HK-2 cells. As demonstrated in Figure 5(A) and (B), the cell viability was inhibited by LPS in a dose- and time-dependent manner (0-100 μg/mL and 0–24 h), and the cell viability was significantly inhibited when exposed to LPS at a concentration of 100 μg/mL for 24 h. Thus, we finally decided to use 100 μg/mL LPS to stimulate HK-2 cells for 24 h in the following *in vitro* experiments. Next, we used the CCK-8 assay to assess the potential cytotoxic effects of XBJ injection at different dilutions (500-, 100-, 50-, 10-, 5-, 2-, and 1-fold) on HK-2 cells and found that XBJ inhibited the viability of HK-2 cells at a 5-flod dilution (Figure 5(C)). Therefore, we injected XBJ at a 10-fold dilution to treat the LPS-stimulated HK-2 cells. XBJ treatment at 10-fold dilution significantly attenuated cellular injury as evidenced by an increase of cell viability (Figure 5(D)), a decrease of cell apoptosis rate (%) (11.31 ± 0.75 *vs.* 8.03 ± 0.58, *p* = 0.004) (Figure 5(E) and (F)), and down-regulated protein expression of C-Cas 3 and Bax along with up-regulated Bcl-2 in HK-2 cells stimulated after LPS (Figure 5(G-1)). Moreover, the ELISA results indicated that XBJ treatment suppressed the inflammation levels *via* decreasing the production of pro-inflammatory cytokines including IL-1β (2.83 ± 0.08 *vs.* 2.12 ± 0.08 pg/mL, *p* < 0.001), IL-6 (3821.00 ± 171.60 *vs.* 2238.00 ± 159.00 pg/mL, *p* < 0.001), and TNF-α (17.52 ± 1.64 *vs.* 9.75 ± 1.04 pg/mL, *p* = 0.002) in HK-2 cells (Figure 5(J–L)). Collectively, these results indicate that XBJ decreased inflammation and apoptosis in LPS-stimulated HK-2 cells.

### XBJ injection mitigated oxidative stress and mitochondrial dysfunction in LPS-stimulated HK-2 cells

**Figure 6.**
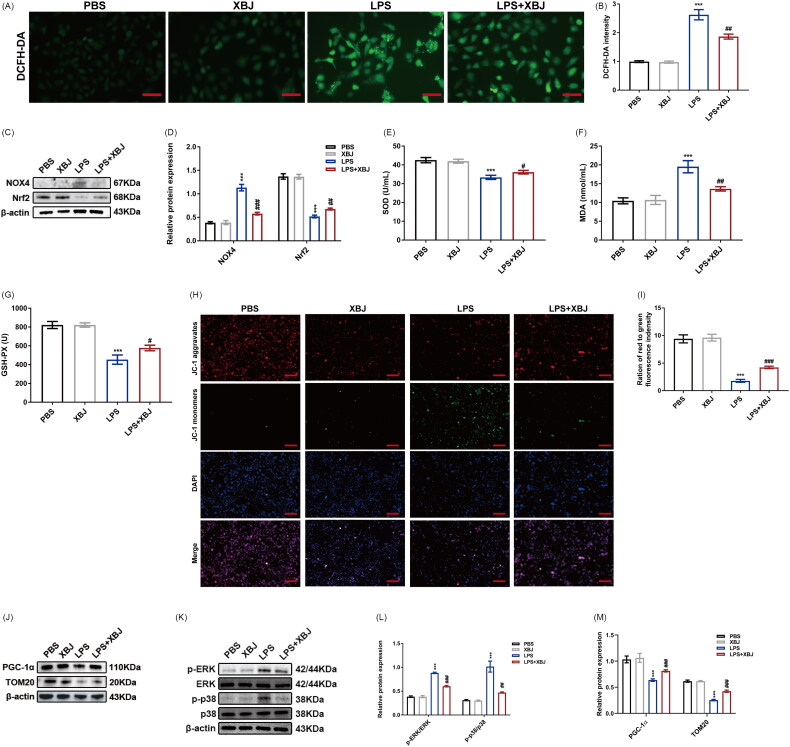
XBJ treatment alleviated mitochondrial dysfunction and inactivated MAPKs in LPS-stimulated HK-2 cells. (A) Representative images of DCFH-DA in HK-2 cells (scale bar = 200 µm) and (B) the quantification of intracellular production of ROS in HK-2 cells (*n* = 3). (C) The protein levels of NOX4 and Nrf2 in HK-2 cells detected by Western blot and (D) quantified by densitometry (*n* = 3). The levels of (E) SOD, (F) MDA, and (G) GSH-Px activity in HK-2 cells (*n* = 5). (H) Representative JC-1 staining images and (I) quantitative analysis of red to green immunofluorescence density (*n* = 3, scale bar = 50 µm). (J) The protein levels of PGC-1α and TOM20 in HK-2 cells detected by Western blot and quantified by densitometry (*n* = 3). (K) The levels of MAPK related proteins (p-ERK, ERK, p-p38, and p38) in HK-2 cells and densitometry quantification (*n* = 3). ***p < 0.001 for LPS versus PBS; ^#^*p* < 0.05, ^##^*p* < 0.01, ^###^*p* < 0.001 for LPS + XBJ versus LPS.

Previous research has indicated that mitochondria play a vital role in maintaining renal function during sepsis [29]. Mitochondrial dysfunction exacerbates oxidative stress, inflammation, and apoptosis, resulting in tubular cell death, forming a vicious loop in renal tubular epithelial cells (RTECs) [30]. Therefore, we evaluated mitochondrial function and detected oxidative stress in subsequent experiments. The immunofluorescence results suggested that XBJ treatment significantly decreased ROS production in LPS-stimulated HK-2 cells (Figure 6(A) and (B)). Western blot analysis confirmed that XBJ decreased NADPH oxidase 4 (NOX4) levels and increased Nrf2 levels in LPS-stimulated HK-2 cells (Figure 6(C) and (D)). Meanwhile, we observed a significant reduction in MDA, associated with an increased production of SOD and GSH-Px, in HK-2 cells treated with LPS + XBJ compared to those treated with LPS alone (Figure 6(E–G)), indicating that XBJ injection can exert antioxidant effects. As the mitochondrial membrane potential is closely linked to oxidative stress and production of ROS [31], we detected the mitochondrial membrane potential by JC-1 staining. XBJ treatment improved the mitochondrial ­membrane potential, as evidenced by an increased ratio of red to green fluorescence (Figure 6(H, 1)). Peroxisome proliferator-activated receptor gamma coactivator 1 alpha (PGC-1α) is a key regulator of mitochondrial biogenesis and translocase of the outer membrane 20 (TOM20) is located in the outer membrane of mitochondria [32]. Therefore, we analyzed the expression of PGC-1α and TOM20 in HK-2 cells by western blot. Compared with PBS control, LPS stimulation decreased the protein levels of PGC-1α and TOM20. Conversely, XBJ treatment increased the PGC-1α and TOM20 levels in LPS-stimulated HK-2 cells (Figure 6(J)). Previous studies have reported that MAPK pathways mediate oxidative stress and mitochondrial dysfunction [22,33]. We measured the protein levels of extracellular signal-regulated kinase (ERK), p-ERK, p38, and p-p38. Western blot demonstrated that XBJ administration suppressed the activation of p-ERK and p-p38 in LPS-stimulated HK-2 cells (Figure 6(K)). In general, XBJ injection suppressed oxidative stress and mitochondrial dysfunction in LPS-stimulated HK-2 cells by repressing MAPK pathways.

### XBJ injection repressed oxidative stress and mitochondrial dysfunction in CLP-injured kidneys

**Figure 7.**
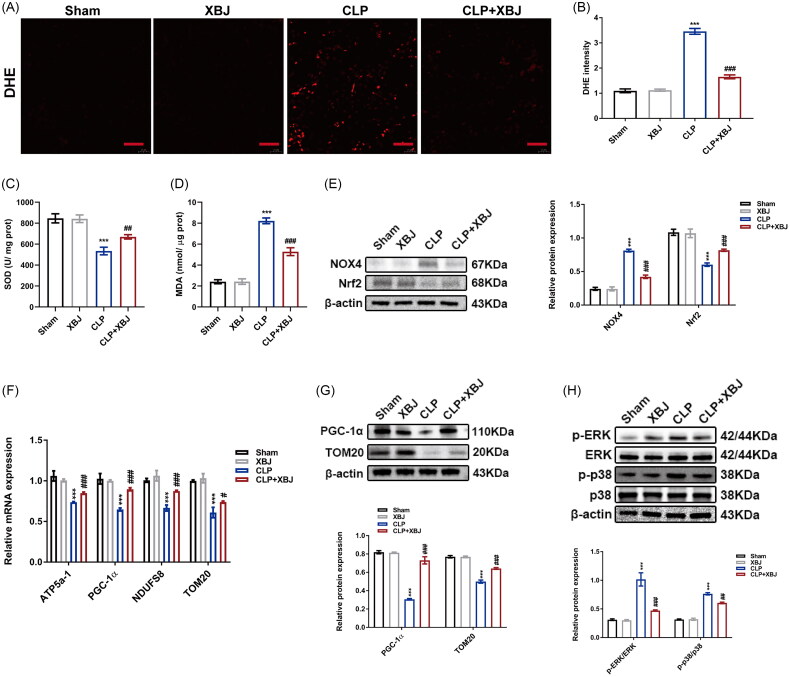
XBJ treatment alleviated oxidative stress in CLP-injured kidneys. (A) Representative images of DHE (scale bar = 20 µm) and (B) the quantification of intracellular production of ROS in kidneys (*n* = 3). The levels of (C) SOD and (D) MDA in kidneys (*n* = 5). (E) The protein levels of NOX4 and Nrf2 in kidneys detected by Western blot and quantified by densitometry (*n* = 3). (F) The mRNA expression levels of ATP5a-1, PGC-1α, NDUFS8, and TOM20 in kidneys (*n* = 3). (G) The protein levels of PGC-1α and TOM20 in kidneys detected by Western blot and quantified by densitometry (*n* = 3). (H) The levels of MAPK related proteins (p-ERK, ERK, p-p38, and p38) in kidneys and densitometry quantification (*n* = 3). ***p < 0.001 for CLP versus sham; ^#^*p* < 0.05, ^##^*p* < 0.01, ^###^*p* < 0.001 for CLP + XBJ versus CLP.

To detect intracellular ROS *in vivo*, DHE was used, and MDA and SOD levels were measured to evaluate the end products of lipid peroxidation. Compared with the sham group, CLP induced ROS production and lipid peroxidation in the kidneys, which were attenuated by XBJ injection (Figure 7(A–D)). CLP-induced upregulation of NOX4 and downregulation of Nrf2 were reversed by XBJ treatment (Figure 7(E)). Furthermore, the reduction of mitochondrial biogenesis-related genes such as ATP5a-1, PGC-1α, NADH: ubiquinone oxidoreductase core subunit S8 (NDUFS8), and protein levels of PGC-1α and TOM20 in CLP-injured kidneys were also reversed by XBJ treatment (Figure 7(F) and (G)). Similarly, western blot revealed that XBJ administration suppressed the activation of p-ERK and p-p38 in CLP-injured kidneys (Figure 7(H)). Overall, the above results indicated that XBJ injection repressed oxidative stress and mitochondrial dysfunction in CLP-injured kidneys by repressing the MAPK pathways.

### XBJ treatment inhibited TLR4/MyD88/NF-κB signaling and ER stress in septic AKI

**Figure 8.**
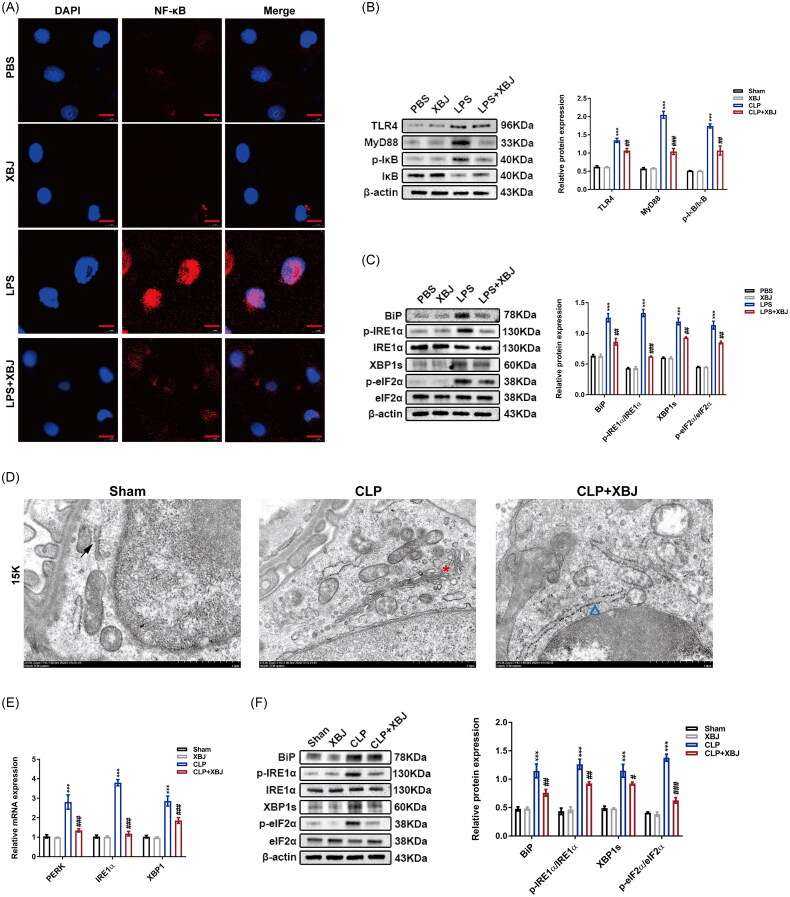
XBJ treatment inhibited TLR4/MyD88/NF-κB axis and ER stress in septic AKI. (A) Representative images of NF-κB fluorescence staining of HK-2 cells (scale bar = 10 µm). (B) The protein levels of TLR4, MyD88, p-IκB, and IκB in HK-2 cells and densitometry quantification (*n* = 3). (C) The protein levels of BiP, p-IRE1α, IRE1α, XBP1s, p-eIF2α, and eIF2α in HK-2 cells and densitometry quantification (*n* = 3). (D) Representative images of ER structure in kidneys captured by TEM (scale bar = 1 µm; black arrow: normal ER; red pentagram: abnormal ER with extensive dilatation, swelling, and degranulation; blue triangle: mildly swollen ER). (E) The mRNA expression levels of PERK, IRE1α, and XBP1 in kidney tissues (*n* = 3). (F) The protein expression of BiP, p-IRE1α, IRE1α, XBP1s, p-eIF2α, and eIF2α in kidneys and densitometry quantification (*n* = 3). ***p < 0.001 for LPS versus PBS or CLP versus sham; ^#^*p* < 0.05, ^##^*p* < 0.01, ^###^*p* < 0.001 for LPS + XBJ versus LPS or CLP + XBJ versus CLP.

TLR4, associated with its downstream mediator myeloid differentiation primary response 88 (MyD88), is activated by LPS and regulates inflammation and apoptosis in RTECs during sepsis [24]. NF-κB complex is crucial for initiating inflammatory response, and translocation of NF-κB into the nucleus regulates the transcription of various downstream pro-inflammatory genes [34]. Thus, we sought to explore whether XBJ could alleviate septic AKI *via* suppressing the TLR4/MyD88/NF-κB axis. Immunofluorescence results suggested that the translocation of NF-κB into the nuclei of HK-2 cells induced by LPS stimulation was markedly suppressed by XBJ treatment (Figure 8(A)). Mechanistically, LPS activated TLR4/MyD88/NF-κB pathway was largely prevented by XBJ treatment in HK-2 cells (Figure 8(B)). The accumulation of unfolded and misfolded proteins in the ER can result in ER stress, further contributing to septic AKI [32]. To confirm this, we observed the ER structure using TEM and detected the protein expression of unfolded protein response (UPR)-related proteins including binding immunoglobulin protein (BiP), inositol-requiring enzyme 1 alpha (IRE1α), p-IRE1α, X-box binding protein 1s (XBP1s), eukaryotic translation initiation factor 2-alpha (eIF2α), and p-eIF2α by western blot. Both UPR-related proteins were over-expressed in the CLP group, and XBJ treatment markedly suppressed their expression (Figure 8(C)). We also observed morphological abnormalities in the ER, including kidney dilatation and degranulation. These pathological changes were abrogated by XBJ administration (Figure 8(D)). Consistently, the LPS-induced up-regulation of UPR-related proteins in HK-2 cells was suppressed by XBJ treatment (Figure 8(E) and (F)). Altogether, XBJ injection ameliorated septic AKI by inhibiting the TLR4/MyD88/NF-κB axis and ER stress.

## Discussion

This study demonstrated that XBJ treatment attenuates septic AKI in a murine model of CLP *in vivo* and in LPS-stimulated RTECs *in vitro*. XBJ suppresses inflammation, inhibits tubular cell apoptosis, alleviates ER stress, and improves mitochondrial function in septic AKI.

XBJ injection is widely used in clinical practice in China as an adjunct therapy for sepsis due to its anti-inflammatory and organ-protective properties [35]. Clinical evidence suggests that XBJ administration can lower sepsis-related mortality, reduce inflammatory cytokine levels, and improve hemodynamic stability [36–38]. However, its precise mechanisms remain unclear, particularly in sepsis-induced organ dysfunction. Some studies have reported that XBJ treatment effectively attenuates sepsis-related complications [16–18]. Our study provides mechanistic insights into its renoprotective effects in septic AKI, further supporting its potential role in broader sepsis management. The results showed a significant increase in the 72- h overall survival rate in mice treated with XBJ compared to those treated with CLP, which is similar to the results of previous studies [18]. Additionally, our results indicated that CLP-induced septic AKI was severe, as revealed by PAS staining, renal function measurements, and the expression of kidney injury markers such as KIM1 and NGAL. XBJ treatment significantly alleviated renal dysfunction, as evidenced by a decrease in tubular injury scores, reduction in Scr and BUN levels, and down-regulation of KIM1 and NGAL.

During sepsis, the recognition of exogenous pathogen-associated molecular patterns (PAMPs) and endogenous damage-associated molecular patterns (DAMPs) by pattern recognition receptors (PRRs) is a pathophysiological process involved in the inflammatory response [4,5]. TLR4 acts as a PRR and is widely distributed in the RTECs. Studies have suggested that TLR4, associated with its downstream molecule MyD88, plays an important role in inflammation during sepsis [24,26,39–42]. Our findings demonstrated that XBJ treatment significantly inhibited LPS-induced upregulation of TLR4 and MyD88 expression. Moreover, XBJ administration suppressed the TLR4-mediated phosphorylation of IκB and markedly repressed the translocation of NF-κB into the nucleus of HK-2 cells, ultimately leading to the reduction of pro-inflammatory cytokines such as TNF-α, MCP-1, IL-6, and IL-1β. This suggests that XBJ treatment significantly alleviated sepsis-induced systemic inflammation in septic AKI.

Mitochondrial dysfunction is a critical factor that should not be overlooked in patients with septic AKI. The impact of oxidative stress on mitochondrial homeostasis is complex and plays a crucial role in intracellular homeostasis, apoptosis, and cell survival [43]. NOX4 activation may increase oxidative stress, leading to mitochondrial damage and dysfunction [30]. Conversely, Nrf2 activation can promote the expression of antioxidant genes, enhance intracellular antioxidant capacity, and aid in maintaining mitochondrial homeostasis [44]. Additionally, the activation of PGC-1α may help restore mitochondrial function, maintain intracellular energy metabolism balance, and reduce oxidative stress-induced mitochondrial damage [45]. P38 MAPK is crucial for oxidative stress and inflammation. Its inhibition may protect cells from oxidative stress-induced damage, prevent mitochondrial dysfunction, and maintain cellular balance [43,46]. Our *in vivo* and *in vitro* studies found that XBJ injection can significantly reduce intracellular oxidative stress levels, improve mitochondrial function, promote biosynthesis, maintain intracellular homeostasis, and reduce kidney injury. This finding further emphasizes the importance of mitochondrial dysfunction in septic AKI and the potential of XBJ injection as a therapeutic intervention.

ER is the main site for protein synthesis, modification, and transportation [47,48]. Various stimuli, such as hypoxia, infection, and drugs, can induce ER stress [49]. The abnormal accumulation of unfolded and misfolded proteins in the ER lumen results in the UPR [50]. The UPR involves three main pathways: IRE1α-XBP1, PERK-ATF4, and ATF6 pathways [51,52]. Several proteins are involved in this process. BiP acts as a molecular chaperone, assisting in the correct folding and degradation of misfolded proteins. IRE1α and XBP1s are crucial regulatory factors in the ER stress response, promoting cell adaptation and survival. Additionally, eIF2α and its phosphorylated form, p-eIF2α, inhibit global protein synthesis in response to stress, helping to alleviate the burden on the ER [53]. However, sustained ER stress may lead to the aberrant activation or dysregulation of these proteins, ultimately affecting normal cellular functions and causing cell damage or death [54]. As a core response in multiple intracellular signaling pathways, ER stress also mediates various pathophysiological processes, including cell metabolism and apoptosis, by coupling with several important pathways, such as the inflammatory response and oxidative stress. Emerging evidence suggests that ER stress and TLR4 signaling form a bidirectional regulatory loop that amplifies cellular injury [55–58]. TLR4 activation in response to LPS and DAMPs induces excessive cytokine production and ROS generation, leading to ER stress activation [59]. Meanwhile, prolonged ER stress further enhances TLR4/MyD88/NF-κB signaling, sustaining inflammation and exacerbating tissue damage [60]. This feedback loop perpetuates inflammation and damage, contributing to AKI progression. Our findings suggest that XBJ disrupts this pathological interplay by simultaneously inhibiting TLR4 signaling and alleviating ER stress, thereby mitigating excessive inflammatory responses and renal tubular cell damage. Various forms of AKI can induce ER stress, including ischemia-reperfusion-induced kidney injury, nephrotoxic drug-induced kidney injury, and sepsis-induced kidney injury. Huang et al. [61] reported that the activation of GRP120 by TUG891 attenuated cisplatin-induced AKI by suppressing ER stress. Moreover, Ferrè et al. [39] suggested that XBP1s was upregulated in renal tubules and further contributed to inflammation and injury in septic AKI. In this study, we observed alterations in ER morphology by TEM in septic AKI, and XBJ treatment reversed these morphological changes in the ER. Moreover, over-expression of UPR-related proteins, including BiP, IRE1, and XBP1, was significantly down-regulated by XBJ treatment. Collectively, these results indicate that XBJ treatment ameliorates septic AKI by suppressing ER stress.

Despite the promising findings, several limitations of this study should be acknowledged. First, the small sample size in the animal experiments may affect reproducibility, necessitating validation in larger cohorts. Second, while our findings suggest that XBJ exerts renoprotective effects *via* the TLR4/MyD88/NF-κB pathway, the absence of genetic knockout models or selective inhibitors limits mechanistic validation. Third, the complex composition of XBJ raises uncertainty about its key bioactive compounds, warranting further fractionation and pharmacokinetic studies. Additionally, species differences must be considered when translating findings from the CLP-induced septic AKI model to humans. While our LPS-stimulated HK-2 cell model supports XBJ’s anti-inflammatory effects, it lacks immune and vascular components, which could be addressed using co-culture systems or organoid models. To minimize confounding factors, we employed randomization, blinded assessments, and standardized protocols. The consistency of our findings across independent experiments and multiple analytical techniques strengthens their reliability. However, further studies are needed to explore XBJ’s long-term efficacy and clinical potential.

## Conclusion

Our study demonstrates that XBJ treatment has a significant therapeutic effect on septic AKI. Through network pharmacological analysis, we identified TLR4 as the core gene involved in the effects of XBJ against septic AKI, with the inflammatory response as the most enriched pathway. Furthermore, both *in vivo* and *in vitro* experiments suggested that XBJ treatment could inhibit apoptosis, inflammation, mitochondrial dysfunction, and ER stress *via* the TLR4/MyD88/NF-κB axis. Our findings provide a promising treatment option for septic AKI and a comprehensive reference for understanding the mechanisms of XBJ injection in septic AKI.

## Data Availability

The data that support the findings of this study are available from the corresponding author S. Chen upon reasonable request.

## Authors’ contirbutions

Lei Zhang: conceptualization, formal analysis, resources, writing-review and editing, funding acquisition; Guangyuan Zhang, Weipu Mao, Si Sun, Shuchun Tao and Nieke Zhang: methodology; Yue Gao: methodology, validation, writing-original draft preparation; Guiya Jiang: validation; Ming Chen: conceptualization, supervision, project administration; Xun Lu: conceptualization, formal analysis, writing-original draft preparation; Shuqiu Chen: conceptualization, formal analysis, resources, writing-review and editing, supervision, funding acquisition. All authors have read and agreed to the published version of the manuscript.
